# Assessing alexithymia across negative and positive emotions: Psychometric properties of the Polish version of the Perth Alexithymia Questionnaire

**DOI:** 10.3389/fpsyt.2022.1047191

**Published:** 2022-11-30

**Authors:** Paweł Larionow, David A. Preece, Karolina Mudło-Głagolska

**Affiliations:** ^1^Faculty of Psychology, Kazimierz Wielki University, Bydgoszcz, Poland; ^2^Faculty of Health Sciences, School of Population Health, Curtin University, Perth, WA, Australia; ^3^School of Psychological Science, The University of Western Australia, Perth, WA, Australia; ^4^Brain and Behaviour Division, Telethon Kids Institute, Perth, WA, Australia

**Keywords:** alexithymia, negative emotions, positive emotions, psychometric properties, psychopathology

## Abstract

The Perth Alexithymia Questionnaire (PAQ) is a 24-item self-report measure of alexithymia. Originally developed in English, it was designed to try to enable more comprehensive (i.e., facet-level and valence-specific) alexithymia assessments. This study aimed to introduce and validate a Polish version of the PAQ. Our sample were 1,008 people (69.44% females, 30.06% males and 0.50% non-binary) aged 18–78 (*M* = 29.69, *SD* = 14.15) from the general community. The PAQ's factor structure was verified with confirmatory factor analysis, and convergent and divergent validity were assessed *via* relationships with other measures of alexithymia and mental health symptoms. Our results indicated strong factorial validity, conforming to the intended subscale structure. As expected, all PAQ subscales correlated in expected directions with another established alexithymia measure, and markers of depression, anxiety, and stress symptoms. The PAQ showed good discriminant validity in terms of measuring an alexithymia construct that was separable from people's current level of distress. Test-retest and internal consistency reliabilities were also good. Overall, the Polish PAQ therefore appears to have strong psychometric properties. Our findings add to a growing body of literature supporting the validity of the PAQ, and the multidimensional nature of the alexithymia construct, across different nations and languages.

## Introduction

The term alexithymia, meaning “no words for emotions” in Greek, was first coined by Nemiah and Sifneos (1) based on their observations of psychiatric patients with psychosomatic disorders. Alexithymia is a multidimensional trait with three core components: difficulty identifying one's own feelings (DIF), difficulty describing feelings (DDF), and externally orientated thinking (EOT; expressed in terms of one rarely focusing on their internal emotional states (2, 3). Alexithymia is established as a transdiagnostic risk factor for a range of psychopathologies, including psychosomatic disorders and affective disorders (4). Indeed, the clinical importance of alexithymia assessment has been emphasized in the recent Diagnostic Criteria for Psychosomatic Research (5), and scholars have long noted that alexithymia can impair the effectiveness of psychotherapy approaches (6). Much of this link between alexithymia and psychopathology appears to be due to the impairing effect that alexithymia has on emotion regulation, whereby people with alexithymia have more difficulty effectively managing their emotional states (4, 7).

The assessment of alexithymia is therefore important. Traditionally, alexithymia has been most commonly assessed using Bagby et al. (8) 20-item Toronto Alexithymia Scale-20 (TAS-20). However, the TAS-20 was not originally designed to assess alexithymia at the facet (i.e., DIF, DDF, EOT) level, and the TAS-20 developers recommend that people use only the total scale score as an overall marker of alexithymia (9), with others noting low reliability in the TAS-20 EOT items if an EOT subscale is extracted (10). Increasingly, researchers and clinicians are interested in examining alexithymia at the facet level (11), a trend that has emphasized the need for more comprehensive assessment tools. Moreover, assessment tools for other emotional constructs are increasingly beginning consider valence in their assessment [i.e., assessing constructs across both negative and positive emotions; see Becerra et al. (12)], and recent data has indicated that people typically report higher levels of alexithymia for negative emotions compared to positive emotions (13).

The Perth Alexithymia Questionnaire (PAQ) was recently introduced to try to enable more comprehensive and valence specific alexithymia assessments (14). Our aim in this study is to introduce and validate a Polish version of the PAQ.

The PAQ is a 24-item self-report measure designed to assess the DIF, DDF and EOT components of alexithymia. A key feature of the PAQ is that valence-specific DIF and DDF subscales can be extracted (i.e., exploring these facets across positive and negative emotions separately). Thus, the PAQ consists of five intended subscales: (1) Negative-Difficulty identifying feelings (N-DIF; 4 items, e.g., *When I'm feeling bad, I get confused about what emotion it is*), (2) Positive-Difficulty identifying feelings (P-DIF; 4 items, e.g., *When I'm feeling good, I can't tell whether I'm happy, excited, or amused*), (3) Negative-Difficulty describing feelings (N-DDF; 4 items, e.g., *When I'm feeling bad, I can't talk about those feelings in much depth or detail*), (4) Positive-Difficulty describing feelings (P-DDF; 4 items, e.g., *When I'm feeling good, if I try to describe how I'm feeling I don't know what to say*) and (5) General-Externally orientated thinking (G-EOT; 8 items, e.g., *I don't pay attention to my emotions*). These five subscales can also be combined into several composite scores, including a total scale score assessing overall alexithymia.

Originally developed in English, several language translations of the PAQ have now been published, including Turkish (15), Iranian (16, 17), and Spanish (18) versions. Factor analyses across language versions have consistently supported a 5-factor structure, conforming to the intended five factors (13, 19, 20). The superiority of this five-factor model, in comparison to simpler models, has therefore supported the value of considering facet-level and valence-specific components in alexithymia assessments. All subscales and the total scale score have also consistently shown high levels of internal consistency reliability (21), though test-retest reliability has not yet been evaluated in a published study.

Comparisons with other measures of alexithymia, emotion regulation difficulties, or psychopathology symptoms have also supported the clinical relevance of PAQ scores. The PAQ correlates highly with the TAS-20, although current evidence from direct comparisons suggest that the TAS-20 appears to assess alexithymia only for negative emotions (20, 21). High levels of alexithymia, as assessed by the PAQ are also associated with more emotion regulation difficulties, and higher levels of psychopathology, as would be expected (14). Crucially, the PAQ also appears to assess a construct that is statistically separable from people's current levels of distress (i.e., discriminant validity). In contrast, amidst concerns that alexithymia measures might actually assess distress levels, some discriminant validity problems have been identified in the TAS-20 (22–24).

Whilst psychometric results with the PAQ have been promising to date, as a recently introduced measure, there is a need to continue evaluating its performance in different settings. There is also presently no Polish version of the PAQ, thus limiting capacity to use it with Polish-speaking populations. The aim of the present study was therefore to introduce a Polish version of the PAQ and to evaluate its psychometric properties (i.e., factor structure, internal consistency reliability, test-retest reliability, convergent and divergent validity). We were also interested in presenting general community group norms to help facilitate the interpretation of PAQ scores, and in evaluating the extent to which PAQ scores might differ across demographic categories.

Based on the theory and past work, we anticipated that the 5-factor structure would be the best factor structure, that the PAQ would correlate highly with another alexithymia measure (the TAS-20), as well as measures of depression, anxiety, and stress symptoms. Based on past findings with the TAS-20 (25), we also anticipated that males may report a higher level of alexithymia on the PAQ than females, and that alexithymia levels would increase with age.

## Materials and methods

### Participants and procedure

The sample consisted of 1,008 Polish-speaking adults (700 females, 303 males and 5 non-binary) with ages ranging from 18 to 78 (*M* = 29.69, *SD* = 14.15) from the general population. Detailed sociodemographic characteristics of the sample are presented in Table 1.

**Table 1:** Sociodemographic characteristics of the study samples.

	**Samples**	**Sociodemographic characteristics**
1	Total sample (females, males and non-binary)	*N* = 1,008. Gender: 700 females, 303 males, 5 non-binary. Age: *M* = 29.69, *SD* = 14.15, min. = 18, max. = 78. Residence: 37.50% lived in large cities (above 100,000 inhabitants), 23.51% in towns (from 20,000 to 100,000), 13.99% in small towns (up to 20,000), 25.00% in villages. Education: 30.95% higher, 55.06% secondary, 4.56% vocational, 9.42% primary. Marital status: 52.18% single, 47.82% in relationships.
2	Females aged 18–29	*N* = 451. Age: *M* = 20.16, *SD* = 20.21, min. = 18, max. = 29. Residence: 28.82% lived in large cities (above 100,000 inhabitants), 26.16% in towns (from 20,000 to 100,000), 16.41% in small towns (up to 20,000), 28.60% in villages. Education: 8.43% higher, 71.18% secondary, 5.54% vocational, 14.86% primary. Marital status: 54.10% single, 45.90% in relationships.
3	Females aged 30–78	*N* = 249. Age: *M* = 47.99, *SD* = 11.33, min. = 30, max. = 78. Residence: 41.37% lived in large cities (above 100,000 inhabitants), 22.09% in towns (from 20,000 to 100,000), 13.25% in small towns (up to 20,000), 23.29% in villages. Education: 66.67% higher, 29.72% secondary, 2.81% vocational, 0.80% primary. Marital status: 32.13% single, 67.87% in relationships.
4	Males aged 18–29	*N* = 215. Age: *M* = 22.58, *SD* = 3.00, min. = 18, max. = 29. Residence: 44.19% lived in large cities (above 100,000 inhabitants), 20.00% in towns (from 20,000 to 100,000), 11.63% in small towns (up to 20,000), 24.19% in villages. Education: 21.40% higher, 63.26% secondary, 4.65% vocational, 10.70% primary. Marital status: 78.60% single, 21.40% in relationships.
5	Males aged 30–73	*N* = 88. Age: *M* = 44.68, *SD* = 10.77, min. = 30, max. = 73. Residence: 54.55% lived in large cities (above 100,000 inhabitants), 21.59% in towns (from 20,000 to 100,000), 9.09% in small towns (up to 20,000), 14.77% in villages. Education: 70.45% higher, 23.86% secondary, 4.55% vocational, 1.14% primary. Marital status: 32.95% single, 67.05% in relationships.

The participants were recruited in the first half of 2022 *via* social networks Facebook and Instagram where there was a link to an online anonymous survey. The Kazimierz Wielki University Scientific Research Ethics Committee approved the study. All respondents provided their informed consent digitally before they answered the questions. Not all respondents completed all the measures to avoid common method bias and stress during filling out the questionnaires.

### Measures

1. The PAQ is a 24-item self-report measure of alexithymia (14). The PAQ consists of five subscales (N-DIF, P-DIF, N-DDF, P-DDF, G-EOT) and several composite scores, including a total scale score. Items are scored on a 7-point scale ranging from 1 (s*trongly disagree*) to 7 (*strongly agree*). Higher scores indicate higher levels of alexithymic traits.
2. The TAS-20 is a 20-item self-report measure of alexithymia (8). The Polish version of the questionnaire was used (26). The TAS-20 was originally designed to provide only a total scale score, but DIF (seven items, e.g., *I am often confused about what emotion I am feeling*), DDF (five items, e.g., *I am able to describe my feelings easily*), and EOT (eight items, e.g., *I prefer to analyse problems rather than just describe them*) subscales are also derived. Items are scored on a 5-point scale ranging from 1 (*strongly disagree*) to 5 (*strongly agree*). There are five reverse-scored items. Higher scores indicate higher levels of alexithymia.
3. The Patient Health Questionnaire-4 (PHQ-4) developed by Kroenke et al. (27) in its Polish version by Larionow and Mudło-Głagolska (28) is a 4-item questionnaire for measuring anxiety and depressive symptoms in the previous 2 weeks. The PHQ-4 has two subscales: anxiety (two items, e.g., *Feeling nervous, anxious or on edge*) and depression (two items, e.g., *Feeling down, depressed, or hopeless*). The overall score of anxiety-depressive symptoms can be calculated. The PHQ-4 uses a 4-point Likert scale from 0 (*not at all*) to 3 (*nearly every day*). Higher scores indicate higher levels of symptoms.
4. The Perceived Stress Scale-4 (PSS-4), developed by Cohen et al. (29) in its Polish version (30), was used for measuring the level of perceived stress during the previous month. The PSS-4 has four items (e.g., *In the last month, how often have you felt that you were unable to control the important things in your life?*), which are due to be evaluated on a 4-point Likert scale from 0 (*never*) to 4 (*very often*). Two items are reverse-scored. Higher score indicates a higher level of perceived stress.

### Analytic strategy

The data were screened for accuracy (minimum and maximum range of each variable). There were no missing data. Statistical analysis was carried out using Statistica (version 13.3), IBM SPSS Statistics (version 28) and R (version 4.2.1). In R the following packages were used: *lavaan* and *semTools* (for confirmatory factor analysis [CFA]), as well as *EFAtools* and *psych* (for exploratory factor analysis [EFA] and reliability analysis).

### Translation of the questionnaire

The original English version of the PAQ was translated into Polish by three independent translators (fluent in both Polish and English) and a common Polish translation of the questionnaire was developed. Then it was translated back into English, and this back translation was compared with the original version of the PAQ. Minor corrections were made, resulting in the final Polish version administered in this study.

### Age and gender differences

Pearson correlations between PAQ scores and age were calculated. Additionally, a series of one-way analyses of covariance (ANCOVAs) were used to investigate the influence of gender and age on PAQ scores.

### Factor structure

The following theoretically informed models were tested: (1) a 1-factor model (all 24 items were specified to load on a “general alexithymia” factor), (2) a 2-factor correlated model comprised of two first-order factors (G-EOT and G-DAF) distinguishing between the attention and appraisal components of alexithymia, (3) a 3-factor correlated model comprised of three first-order factors (G-DIF, G-DDF and G-EOT) corresponding to the 3 facets of alexithymia but not accounting for valence, (4) a 3-factor correlated valence-specific model with three first-order factors (N-DAF, P-DAF and G-EOT) where no distinction was made between the identifying and describing components of appraisal but valence was accounted for, and (5) a 5-factor correlated model that reflected the intended subscale structure of the PAQ (N-DIF, P-DIF, N-DDF, P-DDF and G-EOT as five first-order factors).

A sample size of more than 1,000 participants is generally regarded as excellent for factor analytic studies (31), thus our sample size was appropriate for examination of the 24-item PAQ. CFA with maximum likelihood estimation with robust standard errors and the Satorra-Bentler scaled test statistic was used. Fit was judged based on the following fit index values: root mean square error of approximation (RMSEA), standardized root mean square residual (SRMR), comparative fit index (CFI), and Tucker–Lewis index (TLI). RMSEA and SRMR values below 0.08, and CFI and TLI values greater than 0.9 indicate acceptable fit (32). The factor models were also directly compared using the Akaike Information Criterion (AIC). Lower AIC value indicates better fit (33).

### Internal consistency reliability

Cronbach's alpha coefficients (α) and McDonald's omega values (ω) with 95% confidence intervals were calculated for all PAQ subscales and the total score.

### Test-retest reliability

Intraclass correlation coefficients (ICCs; two-way mixed method with absolute agreement type) were calculated to assess the correlation between PAQ scores at baseline and 1-month follow-up. Paired samples *t*-tests (two-sided *p*) were used to compare PAQ scores between these two time points. The standard error of measurement (SEM) and the minimum detectable change (MDC), with a 95% confidence interval, were calculated. The MDC represents the minimum change that a person must present on questionnaire results to ensure that the observed change is real and is not due to a measurement error (34).

### Convergent and divergent validity

Pearson correlations between PAQ scores and TAS-20, PHQ-4, and PSS-4 scores were examined to assess convergent and divergent validity. Additionally, discriminant validity was evaluated by conducting a second-order exploratory factor analysis (principal axis factoring with direct oblimin rotation) of the five PAQ subscales, the two PHQ-4 subscales and the PSS-4 score. It was expected that the PAQ subscales would load on an alexithymia factor, and the PHQ-4 and PSS-4 subscales on a separate negative affect factor (thus supporting discriminant validity).

### Group norms

Group norms were calculated using the sten scale and based on the data of the whole sample. Sten scores were calculated from Z-scores using the formula: sten = (Z-score × 2) + 5.5 (35).

## Results

Table 2 presents descriptive statistics for all the variables in the study. Skewness scores for the PAQ subscales ranged from 0.24 to 0.90, whereas kurtosis ranged from −1.28 to −0.11 (refer to Supplementary Table 1), indicating the distribution of subscales scores was normal. Age, PHQ-4, PSS-4 and TAS-20 scores were also normally distributed (skewness values ranged from −0.15 to 1.31, and kurtosis ones from −1.22 to 1).

**Table 2:** Descriptive statistics and Cronbach's alpha (α) and McDonald's omega values (ω) with 95% confidence intervals (CI) for the PAQ, PHQ-4, PSS-4 and TAS-20.

**Variables**	**Total sample (females, males and non-binary)**					**Females**			**Males**		
	**α (95%CI)**	**ω (95%CI)**	***N***	***M***	***SD***	***N***	***M***	***SD***	***N***	***M***	***SD***
Age	–	–	1,008	29.69	14.15	700	30.06	15.05	303	29.00	11.87
**PAQ subscales**											
Negative-difficulty identifying feelings	0.89 (0.88; 0.90)	0.89 (0.88; 0.90)	1,008	13.14	7.53	700	13.62	7.65	303	11.88	7.11
Positive-difficulty identifying feelings	0.87 (0.85; 0.88)	0.86 (0.85; 0.88)	1,008	11.05	6.77	700	11.08	6.84	303	10.89	6.60
Negative-difficulty describing feelings	0.90 (0.89; 0.91)	0.90 (0.89; 0.91)	1,008	14.68	7.89	700	15.00	8.04	303	13.76	7.40
Positive-difficulty describing feelings	0.88 (0.87; 0.89)	0.88 (0.87; 0.89)	1,008	12.22	7.10	700	12.15	7.15	303	12.24	6.95
General-externally orientated thinking	0.91 (0.90; 0.92)	0.91 (0.90; 0.92)	1,008	25.45	13.21	700	24.92	13.02	303	26.65	13.60
**PAQ composites**											
General-difficulty identifying feelings	0.92 (0.91; 0.93)	0.92 (0.91; 0.93)	1,008	24.19	13.32	700	24.70	13.52	303	22.78	12.74
General-difficulty describing feelings	0.92 (0.91; 0.93)	0.92 (0.91; 0.93)	1,008	26.90	13.88	700	27.15	14.07	303	26.00	13.31
Negative-difficulty appraising feelings	0.94 (0.93; 0.95)	0.94 (0.93; 0.95)	1,008	27.82	14.84	700	28.63	15.17	303	25.64	13.76
Positive-difficulty appraising feelings	0.93 (0.92; 0.94)	0.93 (0.92; 0.94)	1,008	23.27	13.31	700	23.23	13.51	303	23.14	12.81
General-difficulty appraising feelings	0.96 (0.95; 0.96)	0.96 (0.95; 0.96)	1,008	51.09	26.37	700	51.86	26.92	303	48.78	24.80
Total PAQ score (general alexithymia)	0.96 (0.96; 0.96)	0.96 (0.96; 0.96)	1,008	76.53	36.64	700	76.78	37.65	303	75.43	34.24
**PHQ-4**											
Anxiety symptoms	0.76 (0.73; 0.79)	0.76 (0.72; 0.79)	944	3.39	1.86	645	3.54	1.83	294	3.03	1.89
Depressive symptoms	0.82 (0.79; 0.84)	0.82 (0.79; 0.84)	944	3.20	1.99	645	3.23	1.94	294	3.11	2.08
Overall score of anxiety and depressive symptoms	0.85 (0.83; 0.87)	0.85 (0.84; 0.87)	944	6.59	3.52	645	6.77	3.45	294	6.14	3.65
**PSS-4**											
Stress symptoms	0.79 (0.77; 0.81)	0.80 (0.78; 0.82)	912	8.47	3.78	614	8.71	3.69	293	7.91	3.90
**TAS-20**											
Difficulties identifying feelings	0.83 (0.74; 0.90)	0.84 (0.77; 0.91)	43	20.67	7.29	41	20.44	7.38	2	25.50	2.12
Difficulties describing feelings	0.83 (0.72; 0.90)	0.83 (0.75; 0.91)	43	13.65	5.73	41	13.51	5.65	2	16.50	9.19
Externally orientated thinking	0.62 (0.42; 0.76)	0.64 (0.49; 0.79)	43	19.16	5.37	41	18.71	4.78	2	28.50	10.61
Total TAS-20 score	0.85 (0.77; 0.90)	0.87 (0.81; 0.93)	43	53.49	14.50	41	52.66	13.92	2	70.50	21.92

PAQ, Perth Alexithymia Questionnaire; PHQ-4, Patient Health Questionnaire-4; PSS-4, Perceived Stress Scale-4; TAS-20, Toronto Alexithymia Scale-20.

We conducted a paired *t*-test to compare N-DAF and P-DAF scores in order to examine whether emotion valence (negative or positive) influenced the extent of people's difficulties appraising their emotions. The participants reported significantly more difficulties appraising their negative emotions compared to their positive emotions, *t*_(1007)_ = 14.478, *p* < 0.001, Cohen's *d* = 0.456.

### Age and gender differences

A series of one-way ANCOVAs were conducted to examine whether males and females, and younger and older people differed with respect to the PAQ results (N-DIF, P-DIF, N-DDF, P-DDF, G-EOT, and total PAQ score). Gender was used as the independent variable and entered as age as a covariate. Compared to males, and adjusting for age, females scored significantly higher on N-DIF [*F*_(1,1000)_ = 15.664, *p* < 0.001, partial η^2^ = 0.015] and N-DDF [*F*_(1,1000)_ = 7.898, *p* = 0.005, partial η^2^ = 0.008]. Females' and males' PAQ scores were not significantly different for the P-DIF, P-DDF, G-EOT subscales and the total scale score (*p* > 0.05). Age was a significant covariate for all of these analyses (*p* < 0.001), suggesting that levels of alexithymic traits did differ between younger and older adults. Alexithymic traits were negatively correlated with age (*r* from −0.20 to −0.33, all *p* < 0.001; refer to Supplementary Table 2), indicating that younger adults were significantly more alexithymia than older adults.

### CFA

As expected, the 1-factor, 2-factor and 3-factor (no valence) models were a poor fit to the data (Table 3). The 3-factor (valence) model was a good fit, and the 5-factor model was also a good fit with the best fit index values overall. However, in the 5-factor model analysis, the covariance matrix of latent variables was not positive definite. This is a common situation/issue in CFAs called a Heywood case (36). Subsequently, we analyzed the modification indices, which indicated the need to add three correlated error terms into the 5-factor model. Bollen and Lennox (37) noted that errors are generally independent of one another, although they are possible among items using similar wordings or appearing near to each other on the questionnaire. Based on the modification indices, we added error terms between items 1 and 2, and between items 1 and 4, and between items 4 and 5. We felt adding these error terms was theoretically justifiable because of conceptual and wording similarities between those items, and their addition improved fit index values further. All item factor loadings were strong for the 5-factor model with the three error term correlations added (loadings ≥ 0.67, all *p* < 0.001; refer to Supplementary Table 1).

**Table 3:** Goodness-of-fit indices for the PAQ models (maximum likelihood estimation with robust standard errors and a Satorra-Bentler scaled test statistic; *N* = 1008).

**Model**	***χ^2^*/*df***	**CFI**	**TLI**	**RMSEA (90% confidence interval)**	**SRMR**	**AIC**
1-Factor	3,227.137/252	0.747	0.723	0.137 (0.133; 0.142)	0.088	90,920.108
2-Factor: G-DAF and G-EOT factors	2,105.766/251	0.843	0.827	0.109 (0.104; 0.113)	0.063	89,107.628
3-Factor (no valence): G-DIF, G-DDF and G-EOT factors	2,051.159/249	0.848	0.831	0.107 (0.103; 0.111)	0.062	89,007.428
3-Factor (valence): N-DAF, P-DAF and G-EOT factors	1,227.022/249	0.916	0.907	0.080 (0.075; 0.084)	0.050	87,726.111
* 5-factor model: N-DIF, P-DIF, N-DDF, P-DDF and G-EOT factors	1,069.516/242	0.929	0.920	0.074 (0.070; 0.079)	0.047	87,469.061
5-factor model with three error terms: N-DIF, P-DIF, N-DDF, P-DDF and G-EOT factors	859.292/239	0.948	0.940	0.064 (0.060; 0.069)	0.044	87,122.610

* Heywood case (covariance matrix of latent variables is not positive definite). CFI, comparative fit index; TLI, Tucker–Lewis index; RMSEA, root mean square error of approximation; SRMR, standardized root mean square residual; AIC, Akaike Information Criterion; N-DIF, Negative-Difficulty identifying feelings; P-DIF, Positive-Difficulty identifying feelings; N-DDF, Negative-Difficulty describing feelings; P-DDF, Positive-Difficulty describing feelings; G-EOT, General-Externally orientated thinking; G-DIF, General-Difficulty identifying feelings; G-DDF, General-Difficulty describing feelings; N-DAF, Negative-Difficulty appraising feelings; P-DAF, Positive-Difficulty appraising feelings; G-DAF, General-Difficulty appraising feelings.

The values of estimated correlations between the subscale factors of the 5-factor model with three error term correlations added are shown in Table 4. The estimated correlations between subscales of N-DIF and N-DDF were positive and high (*r* = 0.94, *p* < 0.001), as well as between subscales of P-DIF and P-DDF (*r* = 0.95, *p* < 0.001). Slightly lower correlations were reported between G-EOT and N-DIF, N-DDF, P-DIF as well as P-DDF, which ranged from 0.63 to 0.70 (all *p* < 0.001). Summarizing, the CFA results showed that 3-factor (valence) and 5-factor model with three error terms are optimal solutions, with the 5-factor model being superior in fit statistics. Thus, we selected the 5-factor model with three error terms as the best solution in our data-set, reflecting the intended factor structure of the questionnaire.

**Table 4:** Estimated correlations between the factors (subscales) of the 5-factor PAQ model (all *p* < 0.001).

**PAQ subscales (factors)**	**Negative-difficulty identifying feelings**	**Positive-difficulty identifying feelings**	**Negative-difficulty describing feelings**	**Positive-difficulty describing feelings**
Negative-difficulty identifying feelings	—			
Positive-difficulty identifying feelings	0.82	—		
Negative-difficulty describing feelings	0.94	0.72	—	
Positive-difficulty describing feelings	0.78	0.95	0.78	—
General-externally orientated thinking	0.63	0.68	0.68	0.70

PAQ, Perth Alexithymia Questionnaire.

### Convergent and divergent validity

The relationships between the PAQ scores and other study variables were analyzed (see Supplementary Table 2). In general, the PAQ subscales and total score were significantly highly positively correlated with the other measure of alexithymia (overall TAS-20 score; *r* from 0.60 to 0.78, all *p* < 0.001), stress (*r* from 0.20 to 0.42, all *p* < 0.001), as well as anxiety (*r* from 0.16 to 0.40, all *p* < 0.001) and depressive symptoms (*r* from 0.25 to 0.40, all *p* < 0.001).

Our second-order EFA of the alexithymia subscales (the five PAQ subscales), stress (the PSS-4 score), anxiety, and depressive symptoms (the two PHQ-4 subscales) extracted two factors (i.e., factor 1 “general alexithymia” and factor 2 “stress, anxiety and depressive symptoms”; refer Supplementary Table 3). All the PAQ subscales loaded cleanly on the “general alexithymia factor” (loadings from 0.742 to 0.899) and did not load on the “stress, anxiety and depressive symptoms” factor (loadings from −0.089 to 0.138). Thus, the alexithymia construct, as measured by the PAQ, was statistically separable from one's current level of negative affect.

### Internal consistency reliability

All PAQ subscales/composite scores and the total score showed good internal consistency reliability (α ≥ 0.87 and ω ≥ 0.86).

### Test-retest reliability

Twenty two participants (19 females and 3 males, aged 20–41 (*M* = 23.64, *SD* = 5.51) filled out the PAQ two times with approximately 1 months' interval between each test. ICCs of all the PAQ scores between the two time measurements were high (≥ 0.85) and statistically significant (all *p* < 0.001; refer to Supplementary Table 4). The paired samples *t*-test revealed statistically significant differences on N-DIF, N-DDF, G-EOT, and the total PAQ score between the two time points (score decrease at second time point). However, the differences were lower than MDC, thus these changes cannot be considered meaningful or clinically important, thus supporting the PAQ's test-retest reliability.

### Group norms

Based on the current study data, group norms for PAQ scores were calculated using the sten scale for females and males in two age groups (aged 18–29 and ≥ 30; see Supplementary Tables 5–8). Detailed sociodemographic characteristics of these study samples are presented in Table 1.

## Discussion

The aim of the study was to introduce a Polish version of the PAQ and explore its psychometric properties. Overall, the analyses empirically supported the validity and reliability of the Polish version of the PAQ as a measure of alexithymia. The 5-factor model, corresponding to the PAQ's intended subscale structure, had the best fit to the data. Within each valence domain, N-DIF and N-DDF, as well as P-DIF and P-DDF, were highly correlated with each other, however there was statistical value in separating them. The obtained results are thus in line with the conclusions presented in other validation studies (15–17) regarding the predominance of the intended 5-factor model over other factor solutions. The internal consistency reliability of all five subscales and the total score was high, and test-retest reliability was similarly supported. Our results thus indicate that alexithymia is relatively stable trait that can be robustly measured by the PAQ.

Concurrent validity was also supported. The PAQ subscales were significantly and highly positively correlated with another established measure of alexithymia (TAS-20), as well as significantly associated with markers of stress, anxiety and depressive symptoms. Moreover, the PAQ subscales showed empirically good discriminant validity against the general psychological distress factor. These results are also consistent with other validation studies on the PAQ provided in different cultures (15, 18).

In terms of the importance of emotional valence, participants generally reported significantly more difficulties appraising their negative emotions compared to their positive emotions. Thus, our results are consistent with previous reports noting the utility of accounting for valence when assessing alexithymia (14, 18). As for age and gender differences, our results suggest that younger people have significantly higher levels of alexithymia, and females score higher in the N-DIF and N-DDF subscales, i.e., they have more difficulties appraising negative emotions than males. Given these differences, we provided group norms for the all PAQ subscales and composite scores for females and males in two age groups from the general population of Poland. In future work, these norms may be helpful when comparing alexithymia levels across individuals from the general community and clinical samples.

### Limitations and strengths of the study

The validation study took place in a broad general sample with a wide range of ages, but we did not test the PAQ in clinical or adolescent samples. As such, future work will be needed to test the generalisability of our findings. That said, our study lends further support to the cross-cultural validity of the PAQ, largely mirroring the results of past studies conducted in other languages (14–16, 18). This reflects the strengths of the Polish version of the PAQ and presents a good support for conducting the studies on clinical and adolescent samples. The Polish version of the PAQ appears to be useful tool for comprehensively measuring the multidimensional alexithymia construct.

## Data availability statement

The raw data supporting the conclusions of this article will be made available by the authors, without undue reservation.

## Ethics statement

The studies involving human participants were reviewed and approved by the Kazimierz Wielki University Research Ethics Committee. The patients/participants provided their written informed consent to participate in this study.

## Author contributions

PL: conceptualization, formal analysis, data curation, investigation, methodology, writing, reviewing and editing, supervision, and project administration. DP: writing, reviewing, and editing as well as supervision. KM-G: data curation, investigation, and methodology. All authors approved the final article and agreed to the authorship order.

## Funding

This work was supported by the Kazimierz Wielki University (Bydgoszcz, Poland).

## Conflict of interest

The authors declare that the research was conducted in the absence of any commercial or financial relationships that could be construed as a potential conflict of interest.

## Publisher's note

All claims expressed in this article are solely those of the authors and do not necessarily represent those of their affiliated organizations, or those of the publisher, the editors and the reviewers. Any product that may be evaluated in this article, or claim that may be made by its manufacturer, is not guaranteed or endorsed by the publisher.
