# Supplementary material for: Assessing alexithymia across negative and positive emotions: Psychometric properties of the Polish version of the Perth Alexithymia Questionnaire
Source: Front Psychiatry. 2022 Nov 30;13:1047191. doi: 10.3389/fpsyt.2022.1047191 (PMC9748568; doi:10.3389/fpsyt.2022.1047191)
Supplement: Supplementary file 1 [file Data_Sheet_1.docx]

**Supplementary Table 1**

Descriptive statistics of the PAQ items and subscales as well as standardized item factor loadings from confirmatory factor analysis (maximum likelihood estimation with robust standard errors and a Satorra-Bentler scaled test statistic; *N* = 1008).

| PAQ subscales/items (original / Polish) | *M* | *SD* | Skewness | Kurtosis | Factor loadings (5-factor model with 3 correlated errors terms) |
| --- | --- | --- | --- | --- | --- |
| **Negative-Difficulty identifying feelings** | 13.14 | 7.53 | 0.47 | -1.04 | — |
| 2. When I’m feeling *bad*, I can’t tell whether I’m sad, angry, or scared / Kiedy czuję się *źle*, trudno mi stwierdzić czy odczuwam smutek, złość, czy strach | 3.22 | 2.18 | 0.57 | -1.19 | 0.734 |
| 8. When I’m feeling *bad*, I can’t make sense of those feelings / Kiedy czuję się *źle*, nie potrafię zrozumieć tych uczuć | 3.33 | 2.14 | 0.46 | -1.23 | 0.858 |
| 14. When I’m feeling *bad*, I get confused about what emotion it is / Kiedy czuję się *źle*, jestem zdezorientowana/y i nie wiem, jaką emocję odczuwam | 3.39 | 2.19 | 0.43 | -1.31 | 0.865 |
| 20. When I’m feeling *bad*, I’m puzzled by those feelings / Kiedy czuję się *źle*, nie potrafię uzmysłowić sobie, skąd biorą się moje uczucia | 3.20 | 2.16 | 0.58 | -1.12 | 0.815 |
| **Positive-Difficulty identifying feelings** | 11.05 | 6.77 | 0.90 | -0.11 | — |
| 5. When I’m feeling *good*, I can’t tell whether I’m happy, excited, or amused / Kiedy czuję się *dobrze*, trudno mi stwierdzić czy odczuwam radość, zadowolenie, czy ekscytację | 3.02 | 2.13 | 0.68 | -0.98 | 0.697 |
| 11. When I’m feeling *good*, I can’t make sense of those feelings / Kiedy czuję się *dobrze*, nie potrafię zrozumieć tych uczuć | 2.70 | 2.02 | 0.99 | -0.39 | 0.768 |
| 17. When I’m feeling *good*, I get confused about what emotion it is / Kiedy czuję się *dobrze*, jestem zdezorientowana/y i nie wiem, jaką emocję odczuwam | 2.62 | 1.93 | 1.06 | -0.12 | 0.862 |
| 23. When I’m feeling *good*, I’m puzzled by those feelings / Kiedy czuję się *dobrze*, nie potrafię uzmysłowić sobie, skąd biorą się moje uczucia | 2.71 | 1.95 | 0.98 | -0.32 | 0.824 |
| **Negative-Difficulty describing feelings** | 14.68 | 7.89 | 0.24 | -1.28 | — |
| 1. When I’m feeling *bad* (feeling an unpleasant emotion), I can’t find the right words to describe those feelings / Kiedy czuję się *źle* (odczuwam nieprzyjemną emocję), trudno mi znaleźć odpowiednie słowa, by opisać moje uczucia | 3.80 | 2.21 | 0.15 | -1.45 | 0.768 |
| 7. When I’m feeling *bad*, I can’t talk about those feelings in much depth or detail / Kiedy czuję się *źle*, nie mogę mówić o tych uczuciach dogłębnie ani szczegółowo | 3.84 | 2.30 | 0.14 | -1.54 | 0.798 |
| 13. When something *bad* happens, it’s hard for me to put into words how I’m feeling / Kiedy dzieje się coś *złego*, trudno mi opisać słowami to, jak się czuję | 3.53 | 2.26 | 0.33 | -1.44 | 0.871 |
| 19. When I’m feeling *bad*, if I try to describe how I’m feeling I don’t know what to say / Kiedy czuję się *źle*, to jeśli próbuję opisać, jak się czuję, nie wiem, co powiedzieć | 3.51 | 2.26 | 0.38 | -1.41 | 0.886 |
| **Positive-Difficulty describing feelings** | 12.22 | 7.10 | 0.66 | -0.62 | — |
| 4. When I’m feeling *good* (feeling a pleasant emotion), I can’t find the right words to describe those feelings / Kiedy czuję się *dobrze* (odczuwam przyjemną emocję), trudno mi znaleźć odpowiednie słowa, by opisać moje uczucia | 3.20 | 2.14 | 0.51 | -1.17 | 0.692 |
| 10. When I’m feeling *good*, I can’t talk about those feelings in much depth or detail / Kiedy czuję się *dobrze*, nie mogę mówić o tych uczuciach dogłębnie ani szczegółowo | 3.08 | 2.05 | 0.68 | -0.87 | 0.795 |
| 16. When something *good* happens, it’s hard for me to put into words how I’m feeling / Kiedy dzieje się coś *dobrego*, trudno mi opisać słowami to, jak się czuję | 2.99 | 2.07 | 0.75 | -0.82 | 0.875 |
| 22. When I’m feeling *good*, if I try to describe how I’m feeling I don’t know what to say / Kiedy czuję się *dobrze*, to jeśli próbuję opisać to, jak się czuję, nie wiem co powiedzieć | 2.95 | 2.03 | 0.78 | -0.70 | 0.874 |
| **General-Externally orientated thinking** | 25.45 | 13.21 | 0.57 | -0.65 | — |
| 3. I tend to ignore how I feel / Mam tendencję do ignorowania tego, jak się czuję | 3.70 | 2.20 | 0.18 | -1.42 | 0.727 |
| 6. I prefer to just let my feelings happen in the background, rather than focus on them / Wolę pozostawić moje emocje w tle niż skupiać się na ich odczuwaniu | 3.84 | 2.17 | 0.10 | -1.40 | 0.734 |
| 9. I don’t pay attention to my emotions / Nie zwracam uwagi na moje emocje | 2.81 | 2.04 | 0.83 | -0.68 | 0.781 |
| 12. Usually, I try to avoid thinking about what I’m feeling / Zwykle staram się unikać myślenia o tym, co czuję | 3.22 | 2.13 | 0.52 | -1.14 | 0.808 |
| 15. I prefer to focus on things I can actually see or touch, rather than my emotions / Wolę skupiać się na rzeczach, które mogę realnie zobaczyć lub poczuć, a nie na moich emocjach | 3.52 | 2.04 | 0.35 | -1.16 | 0.725 |
| 18. I don’t try to be ‘in touch’ with my emotions / Nie staram się być „w kontakcie” z moimi emocjami | 3.09 | 2.09 | 0.63 | -0.97 | 0.840 |
| 21. It’s not important for me to know what I’m feeling / Nie jest dla mnie ważne, by wiedzieć, co czuję | 2.43 | 1.94 | 1.22 | 0.20 | 0.724 |
| 24. It’s strange for me to think about my emotions / Zastanawianie się nad moimi emocjami jest dla mnie dziwne | 2.85 | 2.19 | 0.85 | -0.79 | 0.674 |

*Note*. *M* = mean; *SD* = standard deviation; PAQ = Perth Alexithymia Questionnaire.

**Supplementary Table 2**

Pearson correlations between scores on the PAQ and PHQ-4, PSS-4 and TAS-20 as well as age.

| Variables | Age (*N* = 1008) | Anxiety symptoms (PHQ-4; *N* = 944) | Depressive symptoms (PHQ-4; *N* = 944) | Overall score of anxiety and depressive symptoms (PHQ-4; *N* = 944) | Stress symptoms (PSS-4; *N* = 912) | Difficulties identifying feelings (TAS-20; *N* = 43) | Difficulties describing feelings (TAS-20; *N* = 43) | Externally orientated thinking (TAS-20; *N* = 43) | Overall TAS-20 score (*N* = 43) | Total TAS-20 score (*N* = 43) |
| --- | --- | --- | --- | --- | --- | --- | --- | --- | --- | --- |
| Negative-Difficulty identifying feelings (PAQ) | **-0.32***** | **0.40***** | **0.40***** | **0.44***** | **0.41***** | **0.78***** | **0.70***** | 0.29 | **0.78***** | **0.78***** |
| Positive-Difficulty identifying feelings (PAQ) | -0.23*** | 0.29*** | **0.34***** | **0.34***** | **0.32***** | **0.62***** | **0.56***** | **0.36*** | **0.67***** | **0.67***** |
| Negative-Difficulty describing feelings (PAQ) | **-0.32***** | **0.35***** | **0.37***** | **0.39***** | **0.39***** | **0.70***** | **0.73***** | 0.19 | **0.71***** | **0.71***** |
| Positive-Difficulty describing feelings (PAQ) | -0.22*** | 0.26*** | **0.33***** | **0.33***** | **0.32***** | **0.60***** | **0.57***** | **0.32*** | **0.65***** | **0.65***** |
| General-Externally orientated thinking (PAQ) | -0.20*** | 0.16*** | 0.25*** | 0.23*** | 0.20*** | **0.44**** | **0.57***** | **0.42**** | **0.60***** | **0.60***** |
| General-Difficulty identifying feelings (PAQ) | **-0.30***** | **0.37***** | **0.40***** | **0.42***** | **0.40***** | **0.75***** | **0.68***** | **0.34*** | **0.77***** | **0.77***** |
| General-Difficulty describing feelings (PAQ) | -0.29*** | **0.34***** | **0.38***** | **0.39***** | **0.38***** | **0.72***** | **0.72***** | 0.28 | **0.75***** | **0.75***** |
| Negative-Difficulty appraising feelings (PAQ) | **-0.33***** | **0.39***** | **0.40***** | **0.43***** | **0.42***** | **0.76***** | **0.74***** | 0.24 | **0.76***** | **0.76***** |
| Positive-Difficulty appraising feelings (PAQ) | -0.23*** | 0.29*** | **0.35***** | **0.35***** | **0.34***** | **0.62***** | **0.58***** | **0.35*** | **0.67***** | **0.67***** |
| General-Difficulty appraising feelings (PAQ) | **-0.31***** | **0.37***** | **0.40***** | **0.42***** | **0.40***** | **0.75***** | **0.72***** | **0.31*** | **0.78***** | **0.78***** |
| Total PAQ score (general alexithymia) | -0.29*** | **0.32***** | **0.38***** | **0.38***** | **0.36***** | **0.68***** | **0.70***** | **0.37*** | **0.76***** | **0.76***** |

*Note*. PAQ = Perth Alexithymia Questionnaire; PHQ-4 = Patient Health Questionnaire-4; PSS-4 = Perceived Stress Scale-4; TAS-20 = Toronto Alexithymia Scale-20; * *p* < 0.05; ** *p* < 0.01; *** *p* < 0.001. Correlation coefficients above 0.3 are shown in bold. The number of the participants (*N*) who completed each questionnaire was shown in the parentheses near the measures.

**Supplementary Table 3**

Factor loadings from a second-order exploratory factor analysis (principal axis factoring with direct oblimin rotation) of the PAQ subscales, stress, anxiety and depressive symptoms (*N* = 912).

| Variables | Factor 1 “general alexithymia” | Factor 2 “stress, anxiety and depressive symptoms” or “distress factor” |
| --- | --- | --- |
| Negative-Difficulty identifying feelings (PAQ) | **0.806** | 0.138 |
| Positive-Difficulty identifying feelings (PAQ) | **0.856** | -0.002 |
| Negative-Difficulty describing feelings (PAQ) | **0.813** | 0.093 |
| Positive-Difficulty describing feelings (PAQ) | **0.899** | -0.035 |
| General-Externally orientated thinking (PAQ) | **0.742** | -0.089 |
| Anxiety symptoms (PHQ-4) | -0.020 | **0.801** |
| Depressive symptoms (PHQ-4) | 0.021 | **0.840** |
| Stress symptoms (PSS-4) | 0.020 | **0.804** |
| Proportion of total variance (%) | 52.7 | 16.4 |

*Note*. PAQ = Perth Alexithymia Questionnaire; PHQ-4 = Patient Health Questionnaire-4; PSS-4 = Perceived Stress Scale-4. Factor loadings > 0.30 are shown in bold.

**Supplementary Table 4**

The test-retest results for the Polish version of the PAQ (*N* = 22).

| Variables | T_1_ | | | T_2_ | | | Mean difference between T_1_ and T_2_ (SE) | Two-sided *p* (paired samples *t*-test) | ICC (95% CI) | SEM | MDC | MDC_95_ |
| --- | --- | --- | --- | --- | --- | --- | --- | --- | --- | --- | --- | --- |
|  | *M* (*SD*) | Skewness | Kurtosis | *M* (*SD*) | Skewness | Kurtosis |  |  |  |  |  |  |
| Negative-Difficulty identifying feelings | 9.77 (4.31) | 0.89 | 0.52 | 8.41 (4.63) | 1.62 | 2.42 | 1.36 (0.60) | 0.035* | 0.870 (0.665; 0.947 | 1.612 | 2.280 | 4.468 |
| Positive-Difficulty identifying feelings | 9.41 (4.59) | 0.59 | -0.31 | 8.86 (5.68) | 1.12 | 0.04 | 0.55 (0.81) | 0.510 | 0.846 (0.630; 0.936) | 2.028 | 2.869 | 5.623 |
| Negative-Difficulty describing feelings | 9.45 (4.03) | 1.01 | 0.99 | 8.27 (4.45) | 1.56 | 2.99 | 1.18 (0.56) | 0.047* | 0.879 (0.696; 0.951) | 1.478 | 2.090 | 4.096 |
| Positive-Difficulty describing feelings | 9.86 (5.73) | 0.47 | -1.37 | 9.32 (5.72) | 1.19 | 0.41 | 0.54 (0.78) | 0.494 | 0.888 (0.731; 0.953) | 1.915 | 2.708 | 5.308 |
| General-Externally orientated thinking | 18.36 (7.28) | 0.58 | -0.10 | 16.45 (7.06) | 0.71 | -0.65 | 1.91 (0.59) | 0.004** | 0.945 (0.794; 0.981) | 1.681 | 2.377 | 4.660 |
| Total PAQ score (general alexithymia) | 56.86 (19.19) | 0.61 | -0.72 | 51.32 (22.07) | 0.82 | -0.60 | 5.54 (2.08) | 0.014* | 0.926 (0.780; 0.972) | 5.626 | 7.957 | 15.596 |

*Note*. * *p* < 0.05; ** *p* < 0.01; T_1_ = Time 1; T_2_ = Time 2; ICC = intraclass correlation coefficient; CI = confidence interval; SE = standard error; SEM = standard error of measurement; MDC = minimal detectable change; MDC_95_ = minimal detectable change with a 95% degree of confidence.

**Supplementary Table 5**

The current (July 2022) norm groups of the Polish version of the PAQ in the group of females aged 18–29 (*N* = 451).

| N-DIF (*M* = 15.95, *SD* = 7.49) | | N-DDF (*M* = 17.47, *SD* = 7.77) | | P-DIF (*M* = 12.40, *SD* = 7.12) | | P-DDF (*M* = 13.52, *SD* = 7.32) | | G-EOT (*M* = 27.70, *SD* = 13.16) | | N-DAF (*M* = 33.42, *SD* = 14.56) | | P-DAF (*M* = 25.92, *SD* = 13.90) | | Total score (*M* = 87.03, *SD* = 36.62) | |
| --- | --- | --- | --- | --- | --- | --- | --- | --- | --- | --- | --- | --- | --- | --- | --- |
| Raw score | Sten | Raw score | Sten | Raw score | Sten | Raw score | Sten | Raw score | Sten | Raw score | Sten | Raw score | Sten | Raw score | Sten |
| — | 1 | — | 1 | — | 1 | — | 1 | — | 1 | — | 1 | — | 1 | — | 1 |
| 4–6 | 2 | 4–7 | 2 | — | 2 | 4 | 2 | 8–11 | 2 | 8–15 | 2 | 8 | 2 | 24–41 | 2 |
| 7–10 | 3 | 8–11 | 3 | 4–7 | 3 | 5–8 | 3 | 12–17 | 3 | 16–22 | 3 | 9–15 | 3 | 42–59 | 3 |
| 11–14 | 4 | 12–15 | 4 | 8–10 | 4 | 9–11 | 4 | 18–24 | 4 | 23–29 | 4 | 16–22 | 4 | 60–77 | 4 |
| 15–17 | 5 | 16–19 | 5 | 11–14 | 5 | 12–15 | 5 | 25–30 | 5 | 30–37 | 5 | 23–29 | 5 | 78–96 | 5 |
| 18–21 | 6 | 20–23 | 6 | 15–17 | 6 | 16–19 | 6 | 31–37 | 6 | 38–44 | 6 | 30–36 | 6 | 97–114 | 6 |
| 22–25 | 7 | 24–27 | 7 | 18–21 | 7 | 20–22 | 7 | 38–44 | 7 | 45–51 | 7 | 37–43 | 7 | 115–132 | 7 |
| 26–28 | 8 | 28 | 8 | 22–24 | 8 | 23–26 | 8 | 45–50 | 8 | 52–56 | 8 | 44–50 | 8 | 133–151 | 8 |
| — | 9 | — | 9 | 25–28 | 9 | 27–28 | 9 | 51–56 | 9 | — | 9 | 51–56 | 9 | 152–168 | 9 |
| — | 10 | — | 10 | — | 10 | — | 10 | — | 10 | — | 10 | — | 10 | — | 10 |

*Note*. N-DIF = Negative-Difficulty identifying feelings; P-DIF = Positive-Difficulty identifying feelings; N-DDF = Negative-Difficulty describing feelings; P-DDF = Positive-Difficulty describing feelings; G-EOT = General-Externally orientated thinking; N-DAF = Negative-Difficulty appraising feelings; P-DAF = Positive-Difficulty appraising feelings.

**Supplementary Table 6**

The current (July 2022) norm groups of the Polish version of the PAQ in the group of females aged 30–78 (*N* = 249).

| N-DIF (*M* = 9.41, *SD* = 5.95) | | N-DDF (*M* = 10.54, *SD* = 6.46) | | P-DIF (*M* = 8.68, *SD* = 5.54) | | P-DDF (*M* = 9.67, *SD* = 6.10) | | G-EOT (*M* = 19.90, *SD* = 11.13) | | N-DAF (*M* = 19.95, *SD* = 12.11) | | P-DAF (*M* = 18.36, *SD* = 11.26) | | Total score (*M* = 58.21, *SD* = 32.01) | |
| --- | --- | --- | --- | --- | --- | --- | --- | --- | --- | --- | --- | --- | --- | --- | --- |
| Raw score | Sten | Raw score | Sten | Raw score | Sten | Raw score | Sten | Raw score | Sten | Raw score | Sten | Raw score | Sten | Raw score | Sten |
| — | 1 | — | 1 | — | 1 | — | 1 | — | 1 | — | 1 | — | 1 | — | 1 |
| — | 2 | — | 2 | — | 2 | — | 2 | — | 2 | — | 2 | — | 2 | — | 2 |
| 4 | 3 | 4–5 | 3 | 4 | 3 | 4–5 | 3 | 8–11 | 3 | 8–10 | 3 | 8–9 | 3 | 24–34 | 3 |
| 5–7 | 4 | 6–8 | 4 | 5–7 | 4 | 6–8 | 4 | 12–17 | 4 | 11–16 | 4 | 10–15 | 4 | 35–50 | 4 |
| 8–10 | 5 | 9–12 | 5 | 8–10 | 5 | 9–11 | 5 | 18–22 | 5 | 17–22 | 5 | 16–21 | 5 | 51–66 | 5 |
| 11–13 | 6 | 13–15 | 6 | 11–12 | 6 | 12–14 | 6 | 23–28 | 6 | 23–29 | 6 | 22–26 | 6 | 67–82 | 6 |
| 14–16 | 7 | 16–18 | 7 | 13–15 | 7 | 15–17 | 7 | 29–33 | 7 | 30–35 | 7 | 27–32 | 7 | 83–98 | 7 |
| 17–19 | 8 | 19–21 | 8 | 16–18 | 8 | 18–20 | 8 | 34–39 | 8 | 36–41 | 8 | 33–38 | 8 | 99–114 | 8 |
| 20–22 | 9 | 22–25 | 9 | 19–21 | 9 | 21–23 | 9 | 40–44 | 9 | 42–47 | 9 | 39–43 | 9 | 115–130 | 9 |
| 23–28 | 10 | 26–28 | 10 | 22–28 | 10 | 24–28 | 10 | 45–56 | 10 | 48–56 | 10 | 44–56 | 10 | 131–168 | 10 |

*Note*. N-DIF = Negative-Difficulty identifying feelings; P-DIF = Positive-Difficulty identifying feelings; N-DDF = Negative-Difficulty describing feelings; P-DDF = Positive-Difficulty describing feelings; G-EOT = General-Externally orientated thinking; N-DAF = Negative-Difficulty appraising feelings; P-DAF = Positive-Difficulty appraising feelings.

**Supplementary Table 7**

The current (July 2022) norm groups of the Polish version of the PAQ in the group of males aged 18–29 (*N* = 215).

| N-DIF (*M* = 12.29, *SD* = 6.95) | | N-DDF (*M* = 14.20, *SD* = 7.25) | | P-DIF (*M* = 11.24, *SD* = 6.58) | | P-DDF (*M* = 12.58, *SD* = 6.87) | | G-EOT (*M* = 26.60, *SD* = 13.21) | | N-DAF (*M* = 26.49, *SD* = 13.31) | | P-DAF (*M* = 23.82, *SD* = 12.62) | | Total score (*M* = 76.91, *SD* = 33.00) | |
| --- | --- | --- | --- | --- | --- | --- | --- | --- | --- | --- | --- | --- | --- | --- | --- |
| Raw score | Sten | Raw score | Sten | Raw score | Sten | Raw score | Sten | Raw score | Sten | Raw score | Sten | Raw score | Sten | Raw score | Sten |
| — | 1 | — | 1 | — | 1 | — | 1 | — | 1 | — | 1 | — | 1 | — | 1 |
| — | 2 | 4–5 | 2 | — | 2 | — | 2 | 8–10 | 2 | 8–9 | 2 | 8 | 2 | 24–35 | 2 |
| 4–7 | 3 | 6–8 | 3 | 4–6 | 3 | 4–7 | 3 | 11–16 | 3 | 10–16 | 3 | 9–14 | 3 | 36–52 | 3 |
| 8–10 | 4 | 9–12 | 4 | 7–9 | 4 | 8–10 | 4 | 17–23 | 4 | 17–23 | 4 | 15–20 | 4 | 53–68 | 4 |
| 11–14 | 5 | 13–16 | 5 | 10–12 | 5 | 11–14 | 5 | 24–29 | 5 | 24–29 | 5 | 21–26 | 5 | 69–85 | 5 |
| 15–17 | 6 | 17–19 | 6 | 13–16 | 6 | 15–17 | 6 | 30–36 | 6 | 30–36 | 6 | 27–33 | 6 | 86–101 | 6 |
| 18–20 | 7 | 20–23 | 7 | 17–19 | 7 | 18–21 | 7 | 37–43 | 7 | 37–43 | 7 | 34–39 | 7 | 102–118 | 7 |
| 21–24 | 8 | 24–26 | 8 | 20–22 | 8 | 22–24 | 8 | 44–49 | 8 | 44–49 | 8 | 40–45 | 8 | 119–134 | 8 |
| 25–27 | 9 | 27–28 | 9 | 23–26 | 9 | 25–28 | 9 | 50–56 | 9 | 50–56 | 9 | 46–52 | 9 | 135–151 | 9 |
| 28 | 10 | — | 10 | 27–28 | 10 | — | 10 | — | 10 | — | 10 | 53–56 | 10 | 152–168 | 10 |

*Note*. N-DIF = Negative-Difficulty identifying feelings; P-DIF = Positive-Difficulty identifying feelings; N-DDF = Negative-Difficulty describing feelings; P-DDF = Positive-Difficulty describing feelings; G-EOT = General-Externally orientated thinking; N-DAF = Negative-Difficulty appraising feelings; P-DAF = Positive-Difficulty appraising feelings.

**Supplementary Table 8**

The current (July 2022) norm groups of the Polish version of the PAQ in the group of males aged 30–73 (*N* = 88).

| N-DIF (*M* = 10.89, *SD* = 7.42) | | N-DDF (*M* = 12.66, *SD* = 7.67) | | P-DIF (*M* = 10.05, *SD* = 6.61) | | P-DDF (*M* = 11.42, *SD* = 7.13) | | G-EOT (*M* = 26.78, *SD* = 14.58) | | N-DAF (*M* = 23.55, *SD* = 14.66) | | P-DAF (*M* = 21.47, *SD* = 13.18) | | Total score (*M* = 71.80, *SD* = 37.04) | |
| --- | --- | --- | --- | --- | --- | --- | --- | --- | --- | --- | --- | --- | --- | --- | --- |
| Raw score | Sten | Raw score | Sten | Raw score | Sten | Raw score | Sten | Raw score | Sten | Raw score | Sten | Raw score | Sten | Raw score | Sten |
| — | 1 | — | 1 | — | 1 | — | 1 | — | 1 | — | 1 | — | 1 | — | 1 |
| — | 2 | — | 2 | — | 2 | — | 2 | 8 | 2 | — | 2 | — | 2 | 24–25 | 2 |
| 4–5 | 3 | 4–6 | 3 | 4–5 | 3 | 4–6 | 3 | 9–15 | 3 | 8–12 | 3 | 8–11 | 3 | 26–44 | 3 |
| 6–9 | 4 | 7–10 | 4 | 6–8 | 4 | 7–9 | 4 | 16–23 | 4 | 13–19 | 4 | 12–18 | 4 | 45–62 | 4 |
| 10–12 | 5 | 11–14 | 5 | 9–11 | 5 | 10–13 | 5 | 24–30 | 5 | 20–27 | 5 | 19–24 | 5 | 63–81 | 5 |
| 13–16 | 6 | 15–18 | 6 | 12–15 | 6 | 14–16 | 6 | 31–37 | 6 | 28–34 | 6 | 25–31 | 6 | 82–99 | 6 |
| 17–20 | 7 | 19–22 | 7 | 16–18 | 7 | 17–20 | 7 | 38–45 | 7 | 35–41 | 7 | 32–37 | 7 | 100–118 | 7 |
| 21–23 | 8 | 23–26 | 8 | 19–21 | 8 | 21–23 | 8 | 46–52 | 8 | 42–49 | 8 | 38–44 | 8 | 119–136 | 8 |
| 24–28 | 9 | 27–28 | 9 | 22–24 | 9 | 24–27 | 9 | 53–56 | 9 | 50–56 | 9 | 45–51 | 9 | 137–155 | 9 |
| 28 | 10 | — | 10 | 25–28 | 10 | 28 | 10 | — | 10 | — | 10 | 52–56 | 10 | 156–168 | 10 |

*Note*. N-DIF = Negative-Difficulty identifying feelings; P-DIF = Positive-Difficulty identifying feelings; N-DDF = Negative-Difficulty describing feelings; P-DDF = Positive-Difficulty describing feelings; G-EOT = General-Externally orientated thinking; N-DAF = Negative-Difficulty appraising feelings; P-DAF = Positive-Difficulty appraising feelings.
